# Correlation between smokeless tobacco (Gutkha) and biomarkers of oxidative stress in plasma with cardiovascular effects

**DOI:** 10.1016/j.heliyon.2020.e05487

**Published:** 2021-02-12

**Authors:** Fareeda Begum Shaik, G. Nagajothi, K. Swarnalatha, Chitta Suresh Kumar, W. Rajendra, Narendra Maddu

**Affiliations:** a Department of Biochemistry, Sri Krishna Devaraya University, Ananthapuramu, 515003, A.P, India; b Department of Corporate Secretaryship, Queen Mary's College (Autonomous), Chennai, T.N, India; c Division of Molecular Biology, Department of Zoology, Sri Venkateswara University, Tirupati, 517502, A.P, India

**Keywords:** Proteins, Metabolite, Biochemistry, Molecular biology, Toxicology, Nicotine, Cotinine, Reactive oxygen species, Reactive nitrogen species, Cardiovascular risk

## Abstract

Tobacco products are widely consumed around the world in smoking and smokeless tobacco (SLT) forms. Analysis of smokeless tobacco consumption suggested that the effects of nicotine and tobacco-specific N-nitrosamines, the main ingredients of smokeless tobacco are attractive to study because its consumption often results in biochemical changes of plasma parameters and markers of oxidative stress development. Smokeless tobacco users generally consume the most commonly available SLT products like khaleja brand of gutkha and mahak chaini brand of khaini 3–5 times per day. We found a significant increase in plasma glucose levels, total cholesterol, triglycerides, and a significant decrease in high-density lipoprotein (HDL) cholesterol indicative of atherosclerosis risk. We also found that the plasma peroxynitrites (ONOO^−^), nitric oxide (NO), lipid peroxidation (LPO), and protein carbonyls (PCO) levels were significantly elevated. Plasma nicotine and cotinine levels were significantly elevated in study subjects, suggesting that nicotine could be responsible for the oxidative and nitrosative stress indirectly inducing cardiovascular risk. There was a strong correlation of nicotine with reactive oxygen species (ROS), reactive nitrogen species (RNS), cholesterol, and creatinine in exposed smokeless tobacco (gutkha) consumers. These data demonstrate SLT users are at high cardiovascular risk due to nicotine-induced free radicals and oxidative damage.

## 1 Introduction

Smoking and smokeless tobacco products are the two modes of tobacco consumption that been assessed throughout the globe and the practice of SLT is common [1]. The SLT products are used orally or without burned and applied under the cheek, lip, and gums and they emerged as the most popular tobacco products and easily accessible during recent years. The species of *Nicotiana Rustica* is used for the manufacture of smokeless tobacco products and is *Nicotiana Tabacum* is used for smoking tobacco products. The higher concentrations of tobacco-specific N-nitrosamines were reported in *N. Rustica* species than the species of N*. Tabacum* [2]. Although smokeless tobacco products from India are reported as popular harmful tobacco products [3], the specific brands and components of different smokeless tobacco products induced biochemical alterations in plasma are not yet defined. The main ingredients of smokeless tobacco products are nicotine alkaloid, tobacco-specific N-nitrosamines (TSNA), N-nitrosamino acids, volatile N-nitrosamines, formaldehyde, acetaldehyde, hydrocarbons, and heavy metals like polonium-210 are described [4].

The use and prevalence of tobacco is high among young adults of 30 years age comprised maybe, with 12% deaths globally from the use of tobacco and related products and smokeless tobacco use is a safer alternative than continued cigarette smoking [5, 6]. The prevalence of chewing SLT products among young people are 35–45% due to product preference [7]. The prevalence and consumption of SLT has greatly enhanced as an alternative source of nicotine addiction due to increased bans on smoking in indoor and public places [8]. The initiating factors are an unsupported perception of safety, indoor smoking bans, increased social acceptance, relaxation, increased concentration, and diminished hunger for greater consumption of SLT products [9, 10]. Currently, numerous forms of smokeless tobacco of various flavours and types of chewing habits are betel quid, khaini, mawa, pan masala plain, and gutkha [11]. The gutkha products are available in the khaleja and rebel brands in India and khaleja brand is the most preferable which are frequently used. Khaini is available in mahak chaini/khaini brand in the Indian tobacco market.

The development of adverse cardiovascular events like myocardial infarction, stroke, ischemic heart disease, oral cancer, and peptic ulcers may be associated with an increased prevalence and consumption of SLT products [12]. The smokeless tobacco and smoking products could deliver a similar amount of nicotine that enhances the toxicity [13]. The Pan Masala containing Tobacco (PMT) users revealed that decreased activities of the antioxidant enzyme system and increased formation of oxidative stress could lead to the risk of cardiovascular disease, peripheral vascular disease, and hypertension [14]. The brands and flavours of smokeless tobacco are well defined and are responsible for the expansion of cardiovascular disease and reactive oxygen species in every consumer, likely by different chemicals in different SLT products.

In the blood at pH 7.4, 69% of nicotine is ionized, 31% unionized, and <5% binds to plasma proteins [15, 16]. Nicotine absorption occurs at a slower rate but at the mucous membranes continued absorption due to chewing habits [17]. Nicotine has been proposed as a risk factor for accelerated atherogenesis by inducing hyperlipidemia condition, injuring endothelial cells, and/or promoting thrombosis, although the evidence is not conclusive. People with coronary heart disease had thrombosis, constricting coronary arteries, and/or facilitating arrhythmogenesis through the adverse effects of nicotine [14]. It would be of great importance to study the correlations of nicotine and cotinine with markers of cardiovascular disease and oxidative stress. To address this question, the purpose of the present study was to explore the biochemical and biophysical alterations in the plasma due to the chronic consumption of smokeless tobacco products.

## 2 Materials and methods

### 2.1 Chemicals

Nicotine with a purity of ≥99% and cotinine of 98% purity used as standards in the HPLC method. Trichloroacetic acid (TCA), Thiobarbituric acid (TBA), Naphthalene ethylene diamine dihydrochloride (NED), Methanol, Dichloromethane (DCM), Diethyl ether (DEE), Acetonitrile (ACN), Sodium n-Heptane sulphonic acid, 5, 5′-Dithiobis, 2-nitrobenzoic acid (DTNB), α, α, Dipyridyl, Ninhydrin-buffer reagent, Chloroform, Phenol, Folin ciocalteau reagent, Sodium dodecyl sulfate, and Potassium dihydrogen phosphate (KH_2_PO_4_) were purchased from Sigma Aldrich, Bangalore.

### 2.2 Study subjects and study description

The study recruited 90 male volunteers who were all smokeless tobacco users (Gutkha and khaini users) and normal healthy controls was performed aged between 20-40 years residing in Ananthapuramu town, Andhra Pradesh, India. This was further divided into three groups of 30 individuals. All the subjects were included smokeless tobacco users for at least four years. The commonly available smokeless tobacco products are chewing 3–5 times per day at 3–10 g each time.

The inclusion criteria are the habitual use of only gutkha and khaini packets by the gutkha and khaini users respectively, and the unmarried and low economic status people. Participants who reported consuming alcohol and or were active smokers were excluded from the study. In the present study, all volunteers were free from any chronic disease, illness, and teetotallers with no smoking habit with free from the use of any tranquilizers, drugs, and anaesthetics. All experiments were performed in accordance with the approved guidelines and regulations of the Ethical Committee (No.25/1/2019-AWD). Blood samples from overnight fasting subjects were used for the study.

Group I: Gutkha users

Group II: Khaini users

Group III: Controls

### 2.3 Collection of blood samples and analysis

Blood samples, drawn from human male volunteers by vein puncture between 7 and 10 AM into heparinized test tubes, were used immediately for plasma analysis. Plasma clinical parameters (Glucose, hemoglobin, total proteins, albumins, glycosylated hemoglobin, cholesterol, triglycerides, HDL-C, liver marker enzymes, and kidney markers) were estimated by auto analyzer kit methods. Plasma iron was estimated by Ramsay, 1958 [18], total amino acids by Moore and Stein, 1948 [19], glycolipids by Roughan and Batt, 1968 [20], total phospholipids by Connerty et al. 1961 [21], Plasma lipid peroxidation was analyzed by the Buege and Aust, 1978 [22], peroxynitrites by the Beckman et al., 1992 [23], protein carbonyls by the Levine et al., 1990 [24], and nitric oxide (NO_2_ and NO_3_) by the Sastry et al., 2002 [25].

### 2.4 Total nitrites (NO_2_) and nitrates (NO_3_)

0.1 mL of plasma samples were treated with 30% zinc sulphate for deproteinization followed by centrifugation at 6, 000 rpm for 5 min. 1.0 mL of the supernatant were swirled for 90 min separately with activated cadmium granules for the conversion of nitrite to nitrate and then griess reagent was added. Nitrite concentrations were estimated using a standard curve developed with sodium nitrite. The nitric oxide was evaluated by the sum of values of nitrites and nitrates.

### 2.5 Lipid peroxidation

The lipid peroxidation was measured by the formation of thiobarbituric acid reacting substances (TBARS). The 2 mL of 15% w/v trichloroacetic acid, 0.375% w/v thiobarbituric acid, and 0.25 N HCl was added to 1 mL plasma sample and kept in boiling water bath for 15 min and then centrifuged at 2, 000 rpm for 10 min. The supernatant was collected and the absorbance was read at 535 nm against the reagent blank assuming the molar extinction coefficient to be 1.56 × 10^5.^

### 2.6 Peroxynitrites

The plasma sample was added to phenol in 50 mM sodium phosphate buffer (pH 7.4) mediated nitration of phenol after incubation for 2 h at 37 °C and NaOH was added and read absorbance at 412 nm. The yield of nitrophenol was calculated from 4400 M^−1^ cm^−1^ as an index of peroxynitrite concentrations.

### 2.7 HPLC

HPLC system (Shimadzu, Japan) is equipped with a binary gradient system with a variable UV/VIS detector (SPD-20A) and a Rheodyne injector with a 20 μL loop and LC-20AD pumps and integrator. Reversed-phase chromatographic analysis was performed in isocratic conditions using a C18 reverse-phase column (Super coil LC-18; 25 cm × 4.6 mm ID; 5μm particle size; 100A° Pore size; Phase-ODS) at 37 °C.

### 2.8 HPLC operating conditions

The resolution of peaks was performed with the mobile phase consisting of a mixture of 0.272 g of KH_2_PO_4_, 0.184 g of sodium n-heptane sulfonate, 820 mL of water, and 180 mL of methanol (HPLC grade). The pH of the mobile phase pH = 3.2 and the flow rate was 1.0 mL/min, and the wavelength was fixed at 256 nm for nicotine and 262 nm for cotinine as per the modified method [26]. Nicotine and cotinine at the concentration of 20 μM/mL were used as standards.

### 2.9 Sample analysis for HPLC

Plasma sample analysis was processed by the modified method [26]. A 100 μL of plasma was alkalinized with 20 μL of 5.0M NaOH, and vortexed. An equal amount of dichloromethane-diethyl ether (1:1 v/v) was used for one-step single extraction, and then vortexed. After centrifugation at 3500 rpm for 3 min, the organic layer was transferred and then evaporated under a stream of nitrogen at 35 °C until dryness and reconstituted in 50 μL of the mobile phase. An aliquot of 20μL was injected into HPLC for analysis.

### 2.10 Statistical data analysis

All the quantitative data are expressed as mean ± SD and one-way ANOVA was used to determine the significance of the parameters between the groups. The Pearson correlation coefficient analyzed using Graph Pad Prism version 6.01 for Windows. *P* < 0.05 was considered statistically significant.

## 3 Results

### 3.1 Levels of glucose, lipid profile, and glycosylated hemoglobin

**Figure 1:**
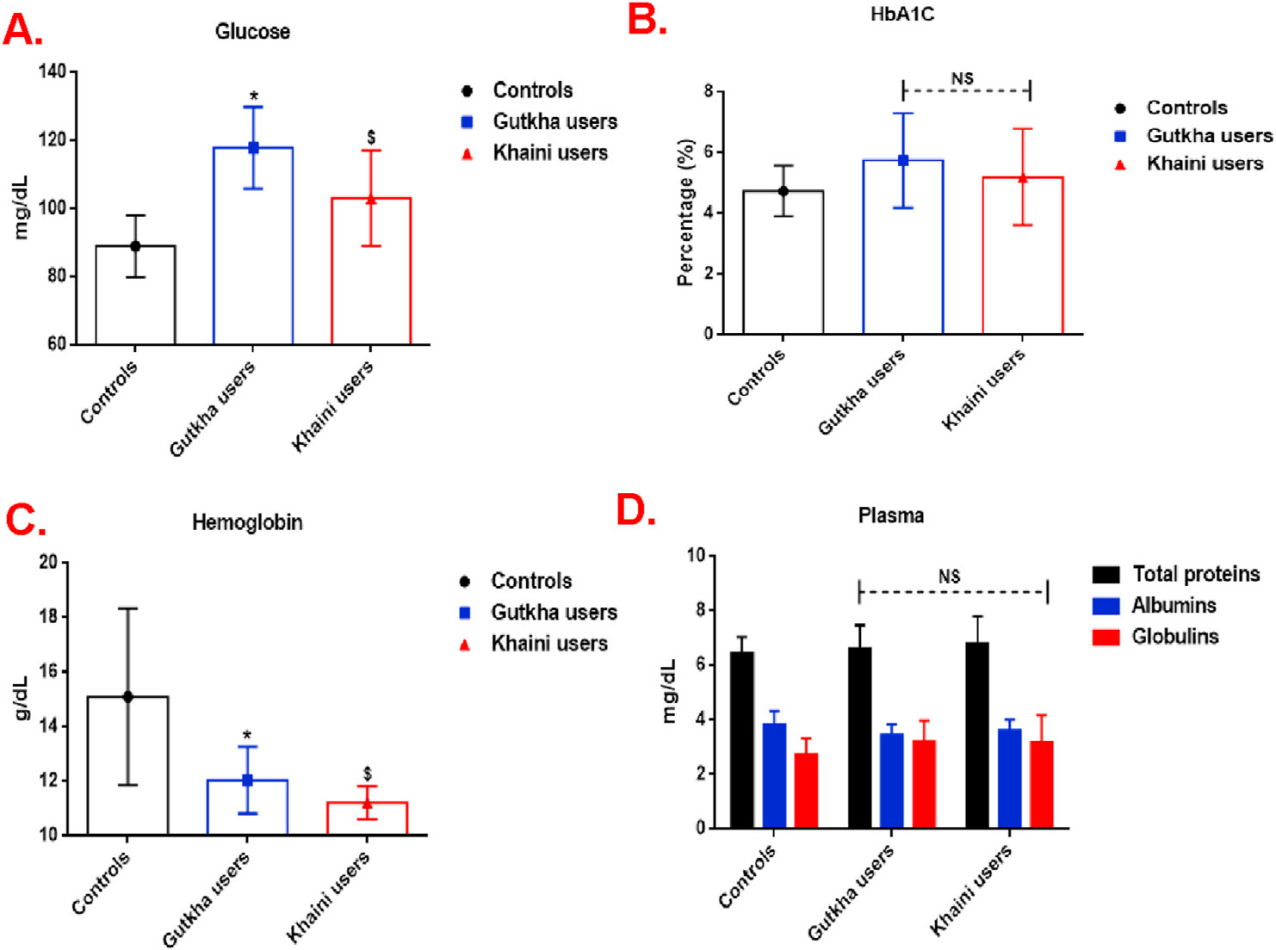
(A, B, C, &D). Biochemical profile in plasma. Data are represented as the mean ± SD. ∗ denotes that data are significantly different from controls and $ denotes that data are significantly different from controls and gutkha groups. Note: NS-Not significant; HbA1C- Glycosylated hemoglobin.

The results of the present study (Figure 1A) indicated that the fasting plasma glucose levels increased significantly in smokeless tobacco users (group I users; +32.41%, group II users; +15.78%) in comparison with the control group (*P* ≤ 0.05). Also, it can be found that increased levels of glycosylated hemoglobin were observed in gutkha and khaini users and there were no significant differences between the groups (Figure 1B). Our data suggested that gutkha and khaini consumers showed significantly decreased levels of blood haemoglobin in comparison with normal control (Figure 1C). Total proteins and globulins were higher in the smokeless tobacco consumers. Plasma albumins showed decreased levels in the study subjects than normal healthy controls (Figure 1D).

**Figure 2:**
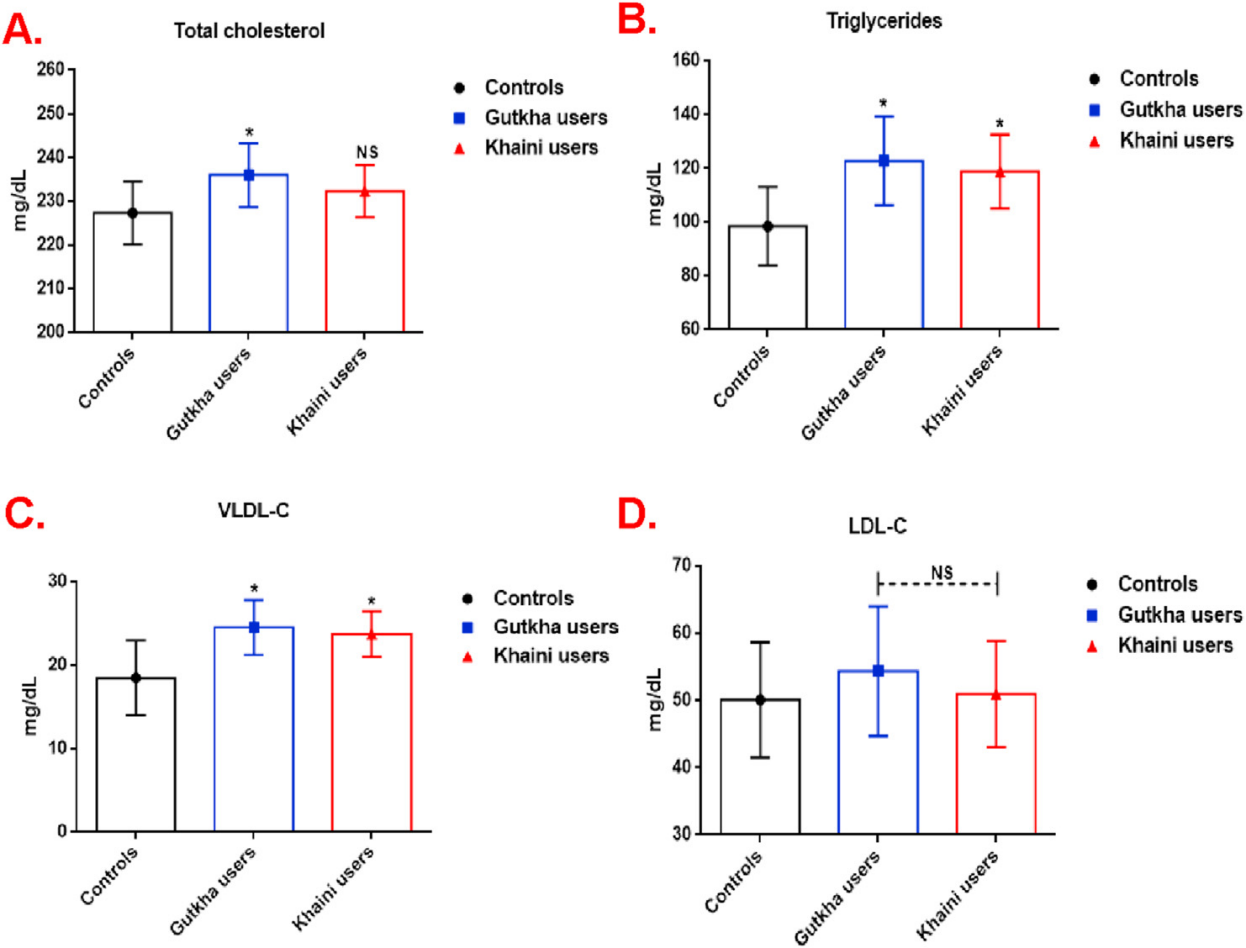
(A, B, C, &D). Lipid and lipoprotein profile in plasma. Data are represented as the mean ± SD. ∗ denotes that data are significantly different from controls. Note: VLDL-C-Very low-density lipoprotein cholesterol; LDL-C-Low-density lipoprotein cholesterol.

The gutkha chewers have shown significantly increased cholesterol levels and khaini chewers showed no significant change of increased cholesterol levels when compared to non-chewers (Figure 2A). More importantly, we observed a significant increase in the levels of triglycerides (+24.74% of gutkha users and +20.66% of khaini users) (Figure 2B) and VLDL-C levels in group I (+32.84%) and group II (+28.47%) consumers in comparison with the control group (Figure 2C). Moreover, it is essential that the higher level of LDL-C was detected in smokeless tobacco users experienced statistically non-significant compared to healthy controls (Figure 2D).

**Figure 3:**
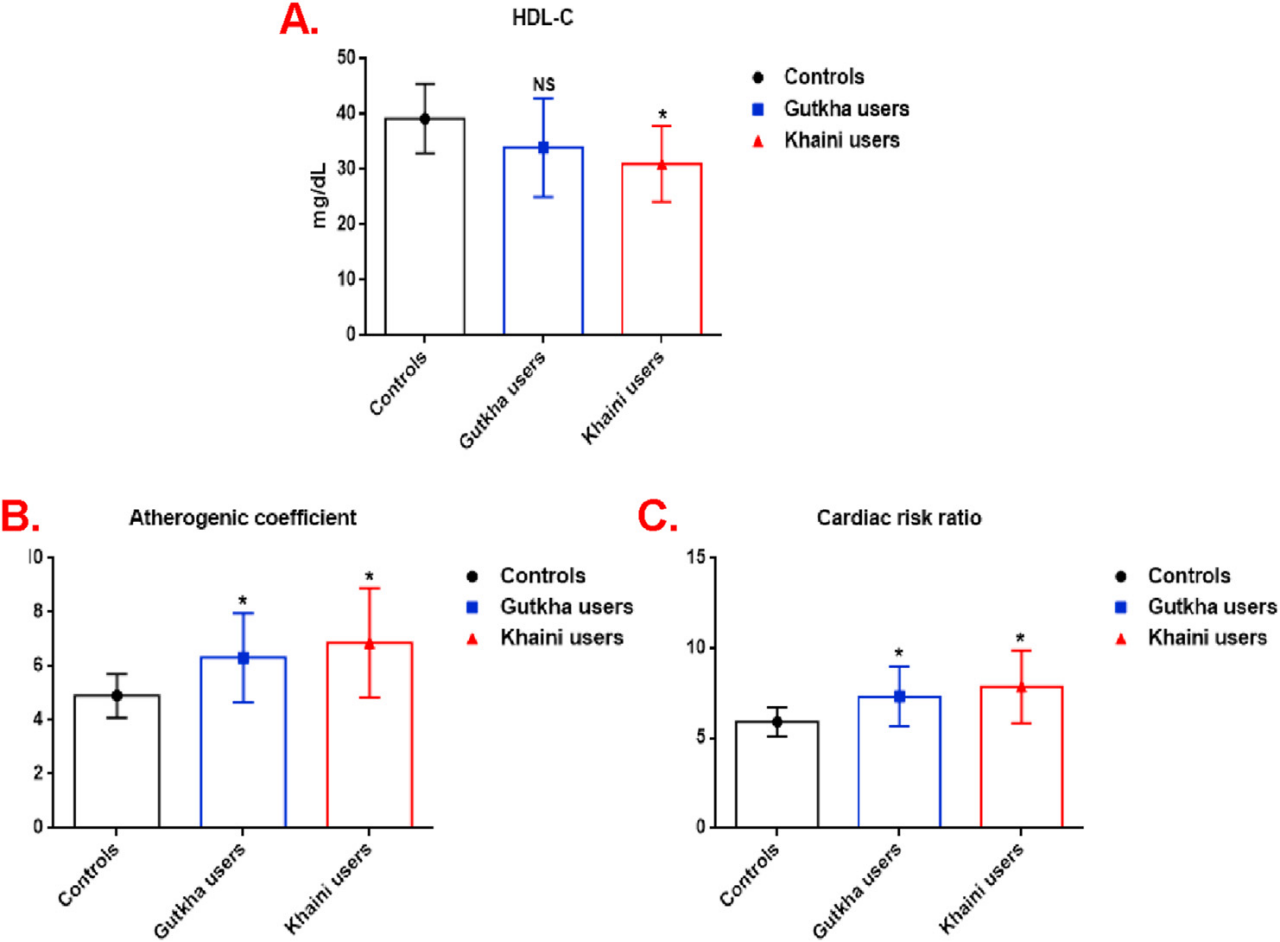
(A, B, &C). Status of HDL-C levels, atherogenic coefficient, and cardiac risk ratio. Data are represented as the mean ± SD. ∗ denotes that data are significantly different from controls. Note: HDL-C- High-density lipoprotein cholesterol.

Our results observed that group II consumers have exhibited a significant decrease of HDL-C levels (-13.39%) and the group I users did not experience statistically significant lower values (-18.23%) than group III users (Figure 3A). A significant difference in increased levels of atherogenic coefficient (Figure 3B) and cardiac risk ratio (Figure 3C) was present in the smokeless tobacco users in comparison with non-SLT users. This information sheds light on the possible lipoprotein profile, glucose concentration in plasma, and status of cardiac risk ratio during smokeless tobacco consumption, as well as on its exposure.

### 3.2 Plasma kidney markers and thiol status

**Figure 4:**
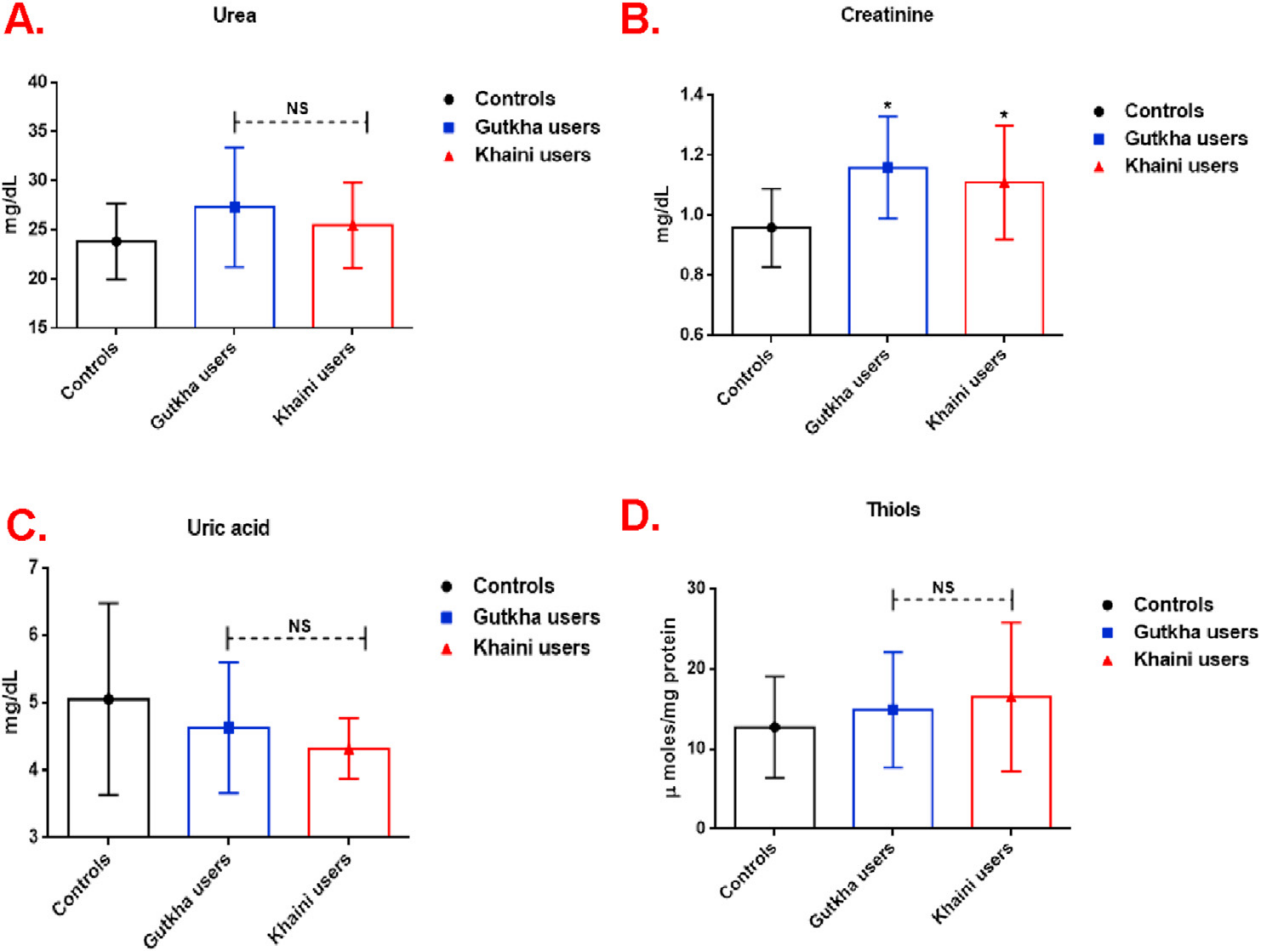
(A, B, C, &D). Levels of kidney markers and thiols in plasma. Data are represented as the mean ± SD. ∗ denotes that data are significantly different from controls.

Data demonstrated (Figure 4A) that the urea levels were found to be increased in study subjects compared to normal controls. Plasma creatinine levels were also increased significantly in smokeless tobacco users (Gutkha users; +20.83% and khaini users; +15.62%) when compared to normal healthy controls (Figure 4B). The mean values of uric acid (Figure 4C) and thiols (Figure 4D) were higher in normal controls did not experience statistically. In the gutkha and khaini users, the significant changes in the concentration of creatinine involved in the biochemical changes in plasma are observed directly.

### 3.3 Concentrations of ROS, RNS, and liver marker enzymes

**Figure 5:**
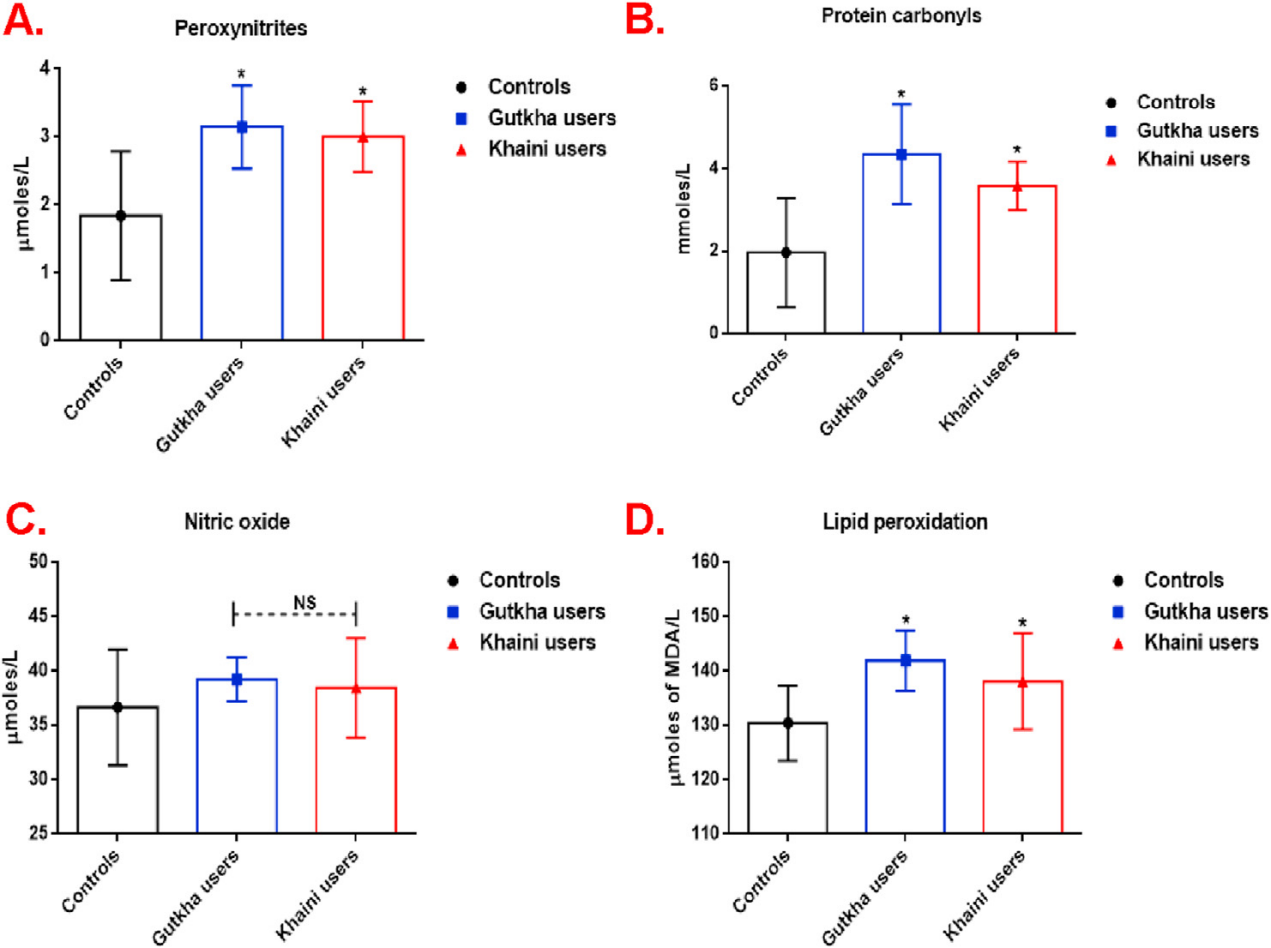
(A, B, C, &D). Levels of nitroxidative stress parameters in gutkha and khaini groups. Data are represented as the mean ± SD. ∗ denotes that data are significantly different from controls. Note: MDA-Malondialdehyde.

**Figure 6:**
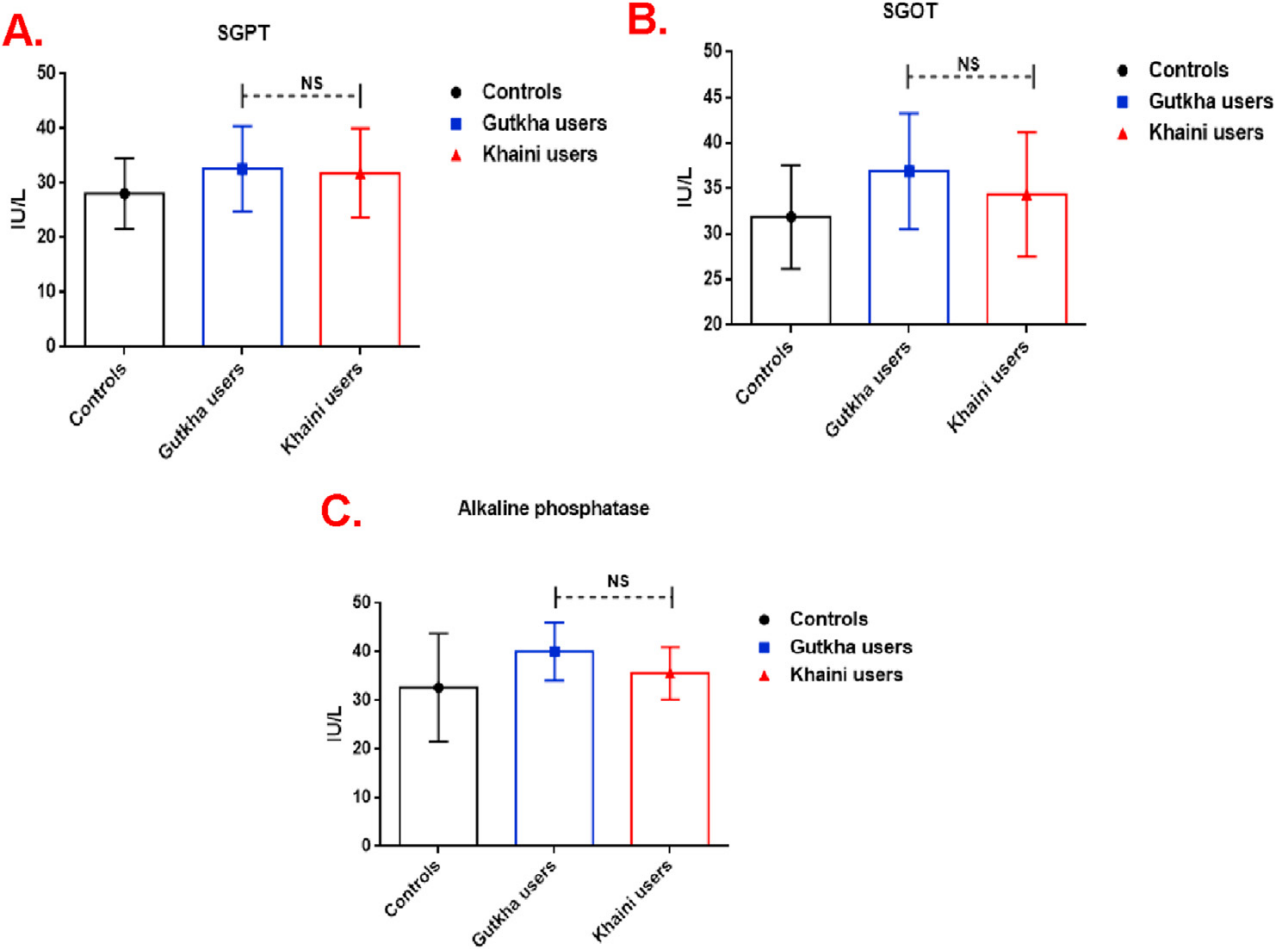
(A, B, &C). Concentrations of liver marker enzymes in controls and study groups. Data are represented as the mean ± SD. ∗ denotes that data are significantly different from controls. Note: SGPT-Serum glutamate pyruvate transaminase; SGOT- Serum glutamate oxaloacetate transaminase.

Our results presented that plasma peroxynitrites levels (group I chewers; +71.19% and group II chewers; +63.58%) and protein carbonyls were significantly increased in group I and group II users compared to group III controls (Figure 5A and 5B). An increased nitric oxide level was present in the plasma of smokeless tobacco users with no significant difference in comparison with non-SLT users (Figure 5C). According to the degree of redox imbalance, the plasma malondialdehyde (MDA) level was significantly increased in gutkha (+8.78%) and khaini groups (+5.84%) than the control group (Figure 5D). These levels of free radicals in the plasma provide a framework for understanding nicotine induced oxidative stress through redox imbalance. The liver marker enzymes like serum glutamate oxaloacetate transaminase (SGOT), serum glutamate pyruvate transaminase (SGPT), alkaline phosphatase (ALP), and levels of thiols were higher in gutkha and khaini chewers compared with non-tobacco users. However, the parameters of SGOT, SGPT, and ALP which was found to be within the normal range showed no significant change (Figure 6A, 6B, and 6C respectively).

### 3.4 HPLC chromatograms

**Figure 7:**
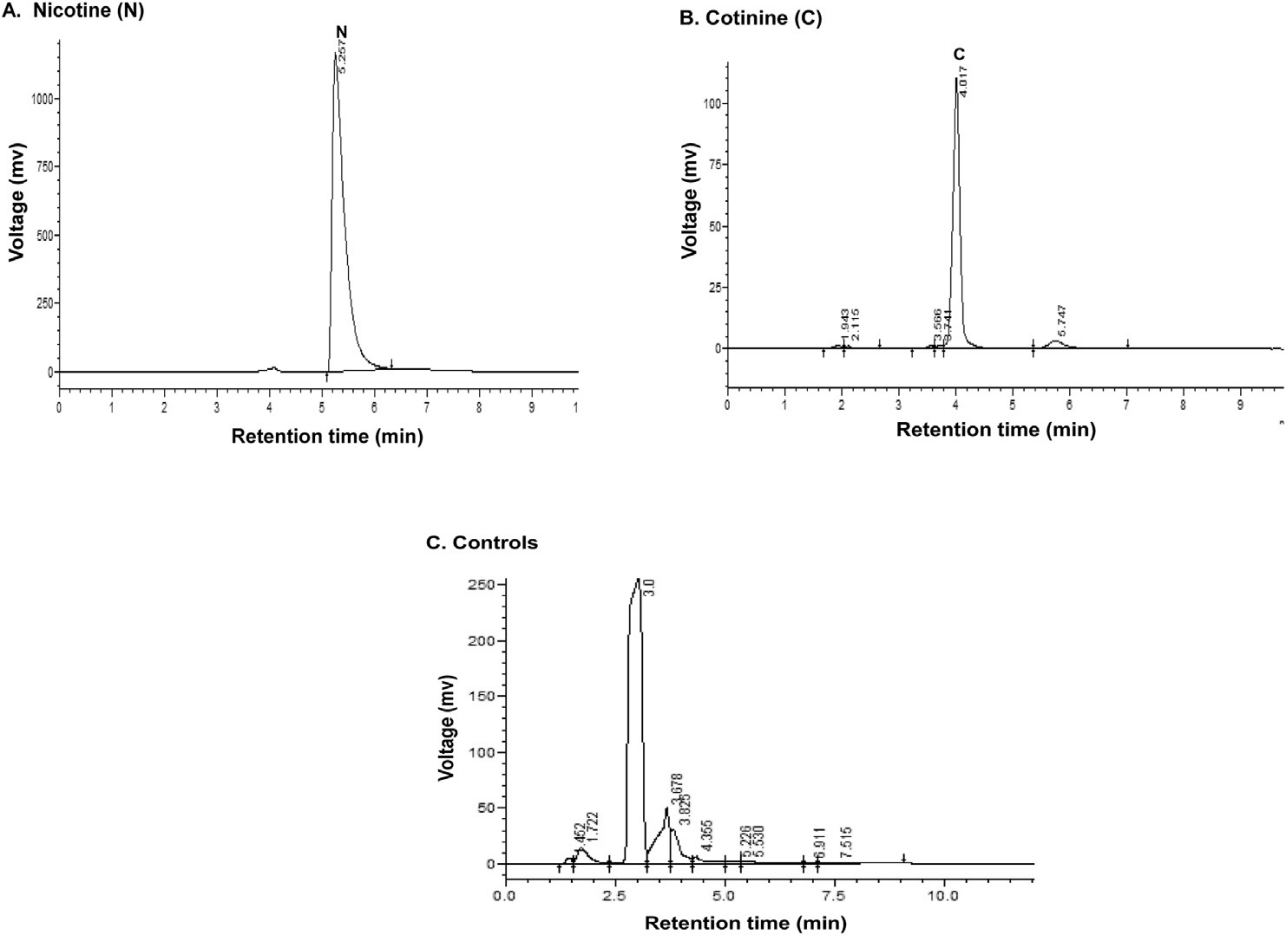
(A, B, &C). HPLC chromatograms of nicotine, cotinine standards, and normal controls.

HPLC analysis showed that the range of retention time of standard nicotine is 5.0–6.0 min and shown a chromatogram peak at 6.51 min (Figure 7A). The range of retention time of standard cotinine is 3.6–4.6 min and showed that a chromatogram peak at 4.01 min (Figure 7B). The normal control group had no nicotine intake and tobacco exposure. Minimal concentrations of nicotine and cotinine levels were observed in the control group due to environmental tobacco exposure and some food constituents. There are no peaks observed in chromatograms of plasma in normal healthy controls at the retention of 4.01 and 5.25 min of nicotine and cotinine (Figure 7C).

### 3.5 HPLC chromatograms in study subjects and concentrations of nicotine and cotinine

**Figure 8:**
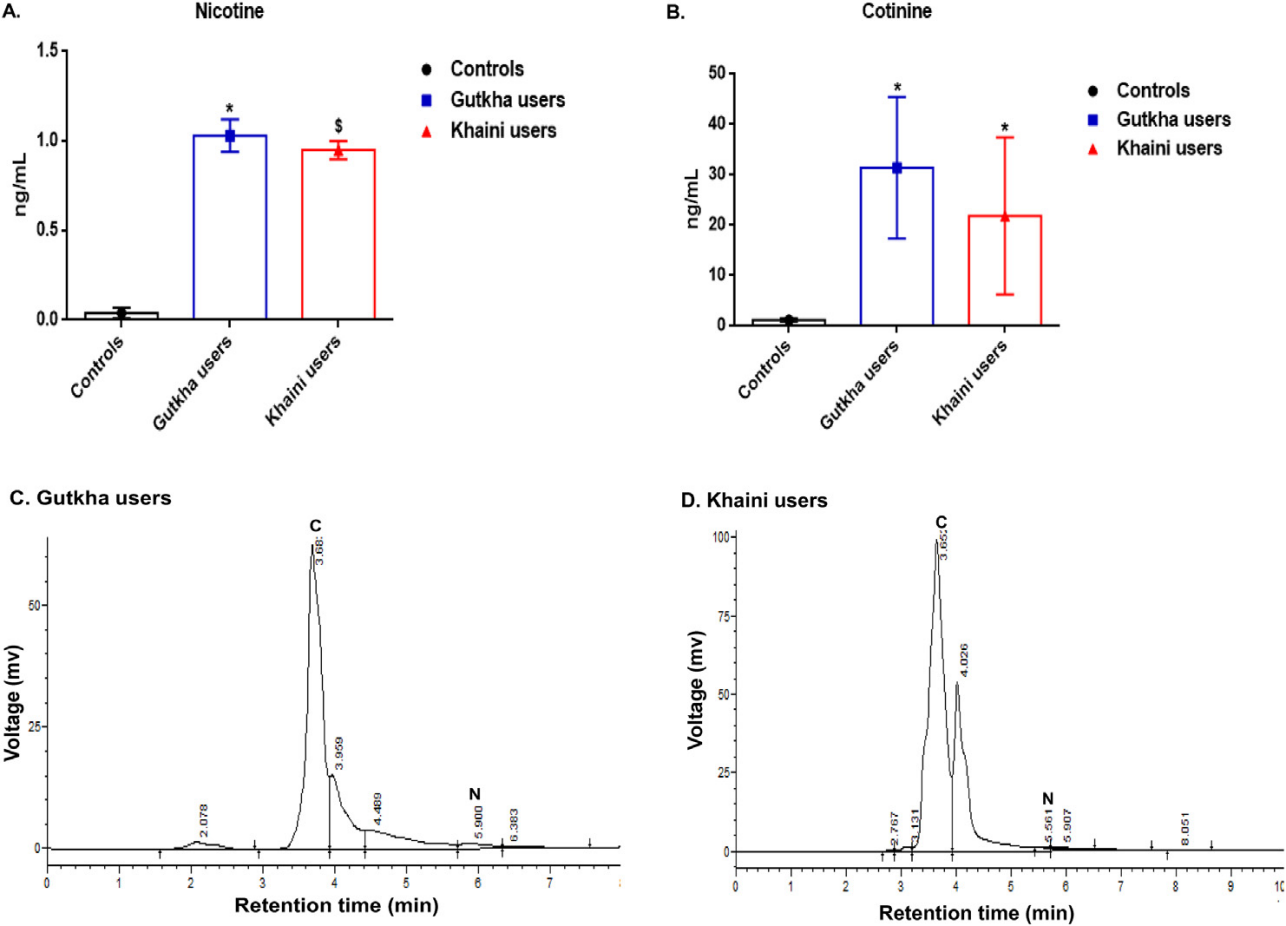
(A, B, C, &D). Plasma concentrations of nicotine, cotinine, and chromatograms in study subjects. Data are represented as the mean ± SD. ∗ denotes that data are significantly different from controls and $ denotes that data are significantly different from controls and gutkha groups.

**Figure 9:**
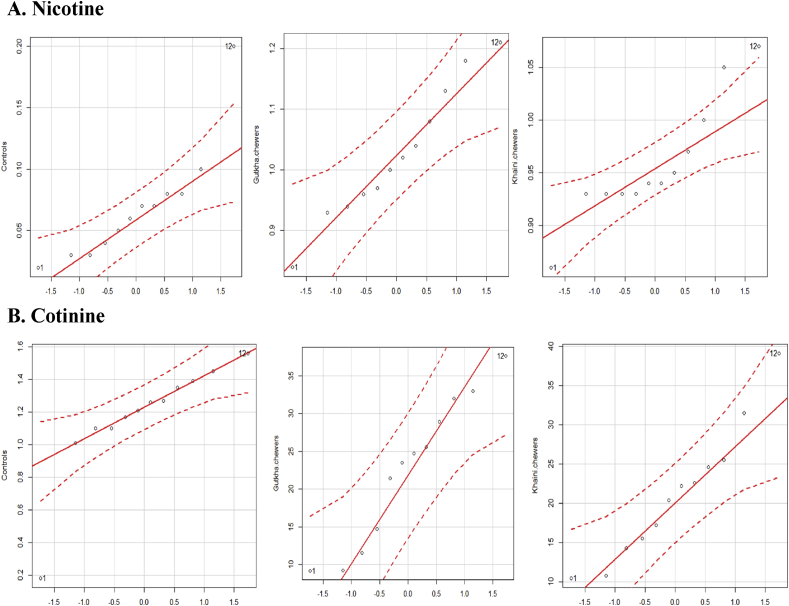
(A &B). Quantile comparison plots of plasma nicotine and cotinine levels.

Smokeless tobacco users showed significantly increased levels of nicotine and cotinine concentrations in plasma of group I and group II users compared to group III controls (Figure 8A and 8B). Gutkha consumers showed that a narrow nicotine chromatogram peak at the retention time of 5.90 min and cotinine peak at 3.60 min. In this group I, a large amount of nicotine is metabolized into cotinine (Figure 8C). Nicotine was shown a chromatogram peak at 5.56 min retention time and cotinine showed that chromatogram peak at 3.65 min in group II consumers (Figure 8D). RP-HPLC technique, therefore, has the capability to directly observe the concentrations of nicotine and cotinine, and thus has the potential to unravel the details of nicotine related pathological disease conditions. The data (Figure 9A and 9B) demonstrated that the distribution of plasma nicotine and cotinine in plasma of controls and smokeless tobacco consumers. Data (Table 1) analysis of HPLC indicated that the highest values of the limit of detection, the limit of quantification were observed for cotinine standard and percentages of recovery test, and precision test were higher reported in standard of nicotine.

**Table 1:** Detection of LOD, LOQ, and precision test from HPLC analysis.

Parameter	Nicotine	Cotinine
Limit of Detection (LOD) (ng/mL)	0.01	0.02
Limit of Quantification (LOQ) (ng/mL)	0.04	0.06
Precision test (%)	57.83	47.35
Recovery test (%)	49.84	32.6

### 3.6 Plasma biochemical profile

A significant increase in concentrations of plasma total phospholipids of gutkha and khaini users with no significant change when compared with non-tobacco users. An increase in levels of total glycolipids observed in non-users and lower levels were reported in study groups with significant change. Smokeless tobacco consumers have described significantly decreased levels of plasma total amino acids in comparison with healthy controls. The level of plasma iron was decreased in gutkha and khaini users showed no significant change compared to normal subjects (Table 2). The concentrations of phospholipids and glycolipids were significant in SLT consumers, which make the process of lipid peroxidation and pro atherogenic risk in a precise manner.

**Table 2:** Smokeless tobacco-induced changes in plasma biochemical profile.

Parameter		Groups	
	Controls	Smokeless tobacco users	
		Gutkha chewers	Khaini chewers
Amino acids (mg/dL)	5.12 ± 0.98	3.72 ± 0.33∗	3.67 ± 0.38∗
Glycolipids (mg/dL)	277.26 ± 19.69	239.08 ± 12.52∗	222.27 ± 14.27^$^
Iron (μg/dL)	127.23 ± 19.15	118.23 ± 10.61^NS^	116.63 ± 12.71^NS^
Phospholipids (mg/dL)	225.30 ± 15.58	239.87 ± 11.23∗	237.45 ± 12.13^NS^

Data are represented as the mean ± SD. ∗ denotes that data are significantly different from controls and $ denotes that data are significantly different from controls and gutkha groups.

### 3.7 Correlation analysis

The correlation analysis have been carried out that gutkha chewers were demonstrated that strong positive correlation of nicotine with plasma biochemical markers except for glucose, hemoglobin which are negatively correlated. In khaini consumers, a strong positive correlation of nicotine with cholesterol and weak positive with creatinine. The negative correlation of HDL-C, hemoglobin and the remaining variables did not experience any correlation in khaini groups. In many cases, the Pearson correlation coefficients were between 0.13 and 0.76 and the values of creatinine, lipid peroxidation, peroxynitrites, protein carbonyls of gutkha users and cholesterol of khaini users exhibited significant difference. We have shown that correlation analysis revealed that gutkha had exerted chronic toxic effects compared to the khaini brand products in human male volunteers (Table 3).

**Table 3:** Correlation of nicotine with plasma biochemical variables.

Nicotine	Gutkha users		Khaini users	
	r	P	r	P
Glucose	-0.16	0.60	-0.05	0.87
Cholesterol	0.55	0.06	0.60	0.036
HDL-C	0.36	0.23	-0.13	0.66
Creatinine	0.67	0.01	0.38	0.21
Hemoglobin	-0.30	0.34	-0.34	0.26
Lipid peroxidation	0.76	0.003	0.073	0.82
Peroxynitrites	0.75	0.004	0.13	0.66
Protein carbonyls	0.65	0.02	0.20	0.51

r = Correlation coefficient; P < 0.05 is considered as statistically significant difference.

These data advance the concept that significant increase in the concentrations of nicotine and cotinine, significantly higher levels of triglycerides, VLDL-C, total cholesterol (No significant change in khaini users), greater levels of protein carbonyls, peroxynitrites, malondialdehyde, and nitric oxide (No significant change in experimental groups), diminished levels of hemoglobin, HDL-C (No significant change in gutkha group), observed in the plasma of gutkha and khaini users compared to normal healthy controls. In addition, certain components like nicotine and tobacco-specific N-nitrosamines continued to remain active during consumption and exert toxic consequences.

## 4 Discussion

The reactive oxygen species production has attracted attention for its potent effects against any pathological disease conditions [27]. There is strong evidence of nicotine in smokeless tobacco has been shown to exert adverse health effects. The food vegetables reported that the minimal amount of nicotine and also present in the air due to environmental tobacco exposure [28]. Significantly increased levels of nicotine and cotinine concentrations were detected in the plasma of group I and group II consumers in comparison to group III controls. Our results agree with those of previous reports revealed that blood nicotine levels were higher in cigarette smokers [29]. Plasma cotinine concentration was used as a very sensitive biomarker for nicotine intake which has been suggested to contribute sensitive changes of a redox system in cells. Cotinine is the active metabolite that appears to be exclusively derived from nicotine and people might be involved in the metabolism of nicotine to cotinine and cotinine to other metabolites may vary differently at different rates [30]. The cotinine levels of plasma were higher in current smokers than non-smokers [31].

Basically, it was shown that nicotine and tobacco-specific nitrosamines could enhance the increased ROS production, and decreased uric acid defense leads to lipid peroxidation and protein oxidation. Likewise, several studies can be found that the various ingredients of smokeless tobacco extract were more toxic than pure nicotine alone in the induction of ROS formation and disparity of redox state [32]. Our recent study has investigated that plasma nitric oxide levels were exhibited to be increased with no significant change in gutkha and khaini users when compared to control subjects. Lime and catechu, used in the preparation of smokeless tobacco products were extensively reported to be involved in the production of ROS and enhance the toxicity persist main etiological threat issues [33]. The enhanced production of nitric oxide through the toxic effects of nicotine leads to endothelial dysfunction [34].

Our results demonstrated that a significant increase in plasma peroxynitrites of smokeless tobacco users in comparison with healthy controls. Upon exposure to the smokeless tobacco components, the red blood cell membrane undergoes functional and structural alterations resulting in increased nitric oxide bioavailability. Moreover, peroxynitrites potency involved in the process of protein nitration, a marker of inducing the atherosclerosis risk has been developed towards this chewing habit due to overall belief against smokeless tobacco [35]. It is of prime importance to note that the direct toxicity of nitric oxide is enhanced through the formation of peroxynitrites from the non-enzymatic reaction of superoxide with nitric oxide. Peroxynitrite is a reactive nitrogen species which become more dangerous and has shown multiple effects like the formation of DNA adducts, process of lipid peroxidation, and protein oxidation [36, 37]. Previous reports have been proposed to be a significantly high serum peroxynitrites level was correlated with serum NO in chronic smokers [38]. It was noticed that peroxynitrites may be involved in the inflammatory responses by reacting with pro-inflammatory interleukins and inducible nitric oxide synthase [36].

Lipid peroxidation, amino acid modifications of proteins, enzyme dysfunction, and DNA damage has been attributed to peroxynitrites [39, 40]. Recently, it was reported that NO may be able to induce DNA damage through the generation of reactive nitrogen species (NO and ONOO-) by the inhibition of DNA damage-repair mechanisms, indirectly involved in the process of carcinogenesis [41]. From the summary statistics, a significant elevation in MDA concentration among smokeless tobacco users when compared to normal subjects. This result is consistent with that obtained by previous studies and malondialdehyde is the most abundant product formed during the lipid peroxidation process and represents a potentially mutagenic and carcinogenic marker [42]. It has been approved that lipid peroxidation indirectly induced atherosclerosis risk by interacting with oxidized LDL [43]. In our present study, a significant increase in fasting plasma glucose levels in the study subjects was observed when compared to controls who do not use smokeless tobacco products and all the study subjects tend to have a normal range of fasting glucose levels.

Cholesterol acts as an essential component of lipoproteins and high levels of cholesterol and triglycerides are the major contributors to atherosclerosis risk as observed in smokeless tobacco consumers. The gutkha and khaini users presented that elevated levels of VLDL-C, LDL-C, and were found to be lower levels of HDL-C are the causative agents for the development of coronary artery disease in which LDL-C acts as bad cholesterol and HDL-C acts as good cholesterol. This result is consistent with LDL-C undergoes oxidation where toxicity was mediated by the formation of oxidized LDL and phospholipids act as both proatherogenic and proinflammatory results in the development of atherosclerosis [44]. It is well known that increased fatty acids are associated with the synthesis of triglycerides and lipoproteins. This is consistent with emerging evidence supported that plasma total cholesterol, LDL-cholesterol, triglyceride levels were determined to be significantly higher and HDL-cholesterol levels were significantly lower in maras powder (SLT) and cigarette smokers than non-tobacco users [45].

Significantly an increased level of total phospholipids in group I and group II chewers was observed in comparison with group III controls. A significant increase in protein carbonyl levels observed in the plasma of smokeless tobacco consumers may cause increased oxidative stress. Creatinine is a useful potential biomarker of renal status and significantly increased levels of creatinine in study subjects and elevated levels of serum urea compared to non-tobacco users and this consequently favors the dysfunction of the renal system. Uric acid levels are decreased in both group I and group II consumers and serve as one of the marker of the formation of reactive oxygen species by acting as a powerful scavenger of free radicals. In addition, it was shown that the uric acid antioxidant regulatory system was affected by nicotine. Our findings highlight the consumption of smokeless tobacco for finding atherosclerosis risk and oxidative stress. Cumulative evidence has suggested that lower serum uric acid levels observed in smokers compared to non-smokers [46].

Nicotine could cause a significant increase in reactive oxygen species levels that resulted in the activation of NF-κB by activating signal responsive kinases and mostly affected [47]. Physiologic nitric oxide concentrations are disseminated locally and by hemoglobin-mediated transport throughout the body which may eventually lead to redox imbalance (Stamler et al., 1997 [48]; Barley et al., 2004 [40]). Significant decrease in hemoglobin concentrations of gutkha and khaini users when compared to non-users. Decreased levels of plasma iron were associated with a significant decrease in blood hemoglobin concentration of smokeless tobacco consumers. Total proteins and globulins are increased in study subjects and several studies have reported that protein content and globulins seem to be slightly increased, indicating that smokeless tobacco does not show much toxicity in the liver [49]. Plasma albumins are decreased in smokeless tobacco consumers due to an antioxidant property of albumin which can be detected by increased oxidative stress. The observed increase in SGOT, SGPT, and ALP levels in the study subjects showed less toxicity to the liver. A significant decrease in plasma total amino acids was associated with an increase in plasma total proteins of smokeless tobacco users compared to non-SLT users.

The correlation analysis revealed significant relationships of nicotine in gutkha with protein carbonyls, peroxynitrites, and lipid peroxidation. Our findings suggest that gutkha may able to induce the process of oxidative stress, through activation and accumulation of lipid peroxidation, increased lipoprotein profile and nitric oxide is widely involved in the progression of ROS related chronic damage to cells as well as other cardiovascular disease conditions is usually due to interplay of several smokeless tobacco products. Other studies indicated that a significant correlation between lipid peroxidation, dropping in non-enzymatic antioxidants, and study subjects was found [50]. The increased concentrations of ROS formation were produced in response to repeated nicotine exposure or pervasive chronic consumption of SLT products and have been faced by the consumers who begin to use these SLT products at an earlier age. We, thus, functionally, the gutkha of smokeless tobacco product than khaini induced specific biophysical and biochemical changes in human male volunteers, including the development of oxidative stress, and cardiovascular disease in the strong correlation of these biomarkers. There is a need for more detailed studies on the relation between specific components of smokeless tobacco and risk of the atherosclerosis through signaling pathways.

## Declarations

### Author contribution statement

N. Maddu: Conceived and designed the experiments; Analyzed and interpreted the data.

C. Kumar: Conceived and designed the experiments.

K. Swarnalatha and S. Begum: Performed the experiments; Wrote the paper.

G. Nagajothi: Analyzed and interpreted the data.

W. Rajendra: Contributed reagents, materials, analysis tools or data.

### Funding statement

S. Begum was supported by an ICMR fellowship during the period of 2013-2018.

### Data availability statement

No data was used for the research described in the article.

### Declaration of interests statement

The authors declare no conflict of interest.

### Additional information

No additional information is available for this paper.
